# Supplementary material for: Multivariate EEG activity reflects the Bayesian integration and the integrated Galilean relative velocity of sensory motion during sensorimotor behavior
Source: Commun Biol. 2023 Jan 28;6:113. doi: 10.1038/s42003-023-04481-2 (PMC9884247; doi:10.1038/s42003-023-04481-2)
Supplement: Supplementary file 2 — Supplementary Information-New [file 42003_2023_4481_MOESM2_ESM.pdf]

Multivariate EEG activity reflects the Bayesian integration and the integrated Galilean relative velocity of sensory motion during sensorimotor behavior

Woojae Jeong[1,4,6], Seolmin Kim[1,2,6], JeongJun Park[1,5], and Joonyeol Lee[*1,2,3]

[1]Center for Neuroscience Imaging Research, Institute for Basic Science (IBS), Suwon 16419, Republic of Korea

[2]Department of Biomedical Engineering, Sungkyunkwan University, Suwon 16419, Republic of Korea

[3]Department of Intelligent Precision Healthcare Convergence, Sungkyunkwan University, Suwon 16419, Republic of Korea

[4]Department of Biomedical Engineering, University of Southern California, Los Angeles, CA 90089, United States of America

[5]Division of Biology and Biomedical Sciences, Program in Neurosciences, Washington University in St. Louis, St. Louis, MO 63130, United States of America

[6]These authors contributed equally to this work: Woojae Jeong, Seolmin Kim.

[*]**Corresponding author:** Department of Biomedical Engineering, Sungkyunkwan University, 2066, Seobu-ro, Jangan-gu, Suwon-si, Gyeonggi-do, 16419, Republic of Korea, Tel: +82 (31) 299-4359, Email: joonyeol@g.skku.edu

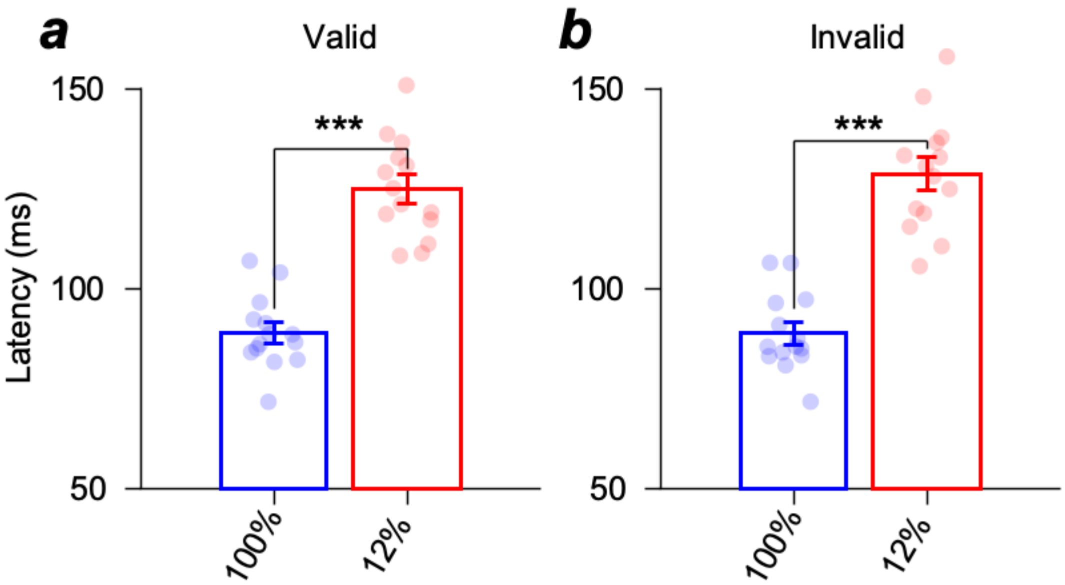

Supplementary Figure 1. Average pursuit latency. a Average pursuit latency in a valid cue condition. **b** Average pursuit latency in an invalid cue condition. Blue bar graphs show the average latency when the contrast of the pursuit target was 100%; red bar graphs show the average latency when the contrast was 12%. The error bars denote the standard errors. $*** \, p < 0.001$.

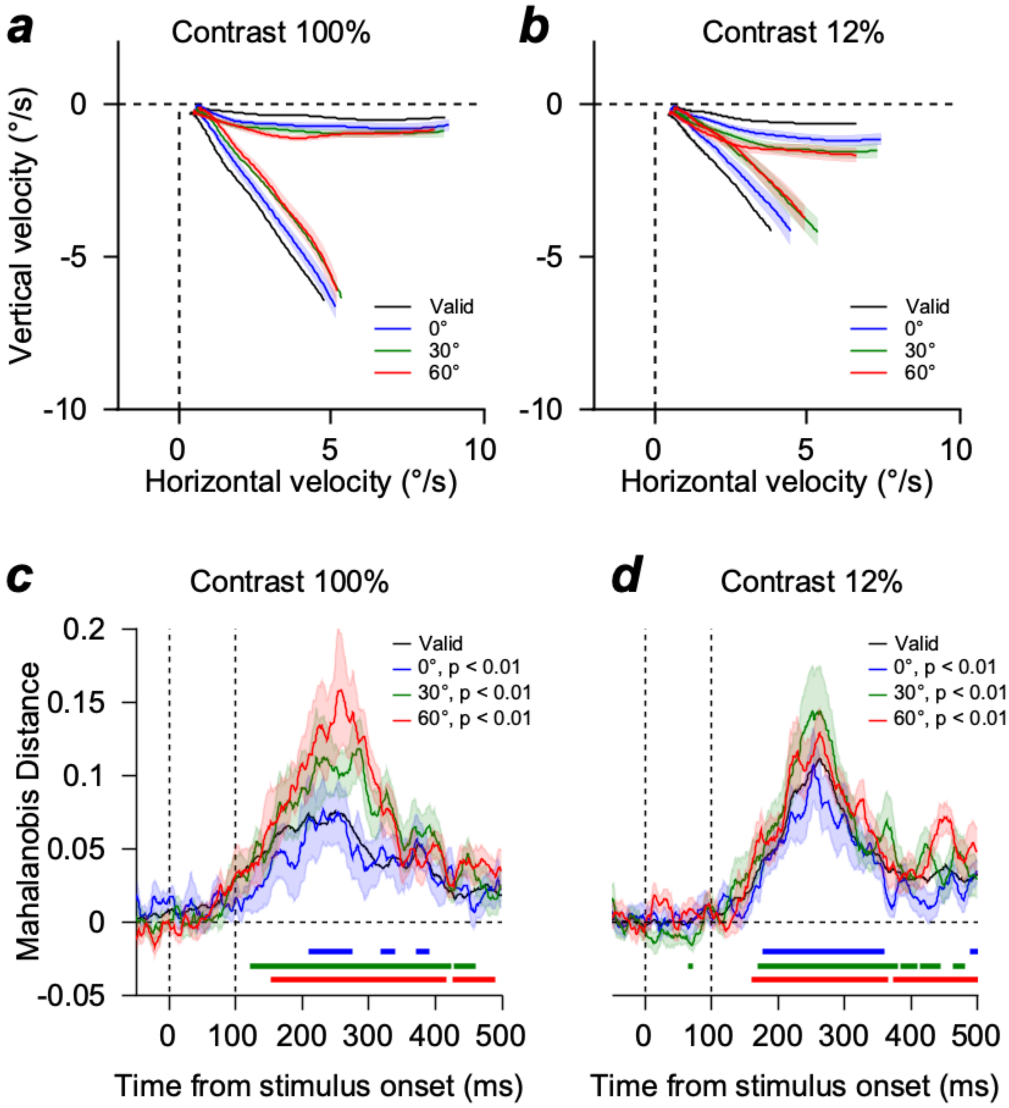

Supplementary Figure 2. Eye movement traces and multivariate EEG pattern dissimilarity (Mahalanobis distance) of invalid blocks for every cue-target condition (difference between cue and target directions = 0°, blue; cue-target = 30°, green; cue-target = 60°, red). The solid black lines show the eye traces (a and b, identical to solid lines in Figure 1c and d) and the EEG pattern dissimilarities (c and d, identical to solid lines in Figure 2a and b) of valid blocks. **a, b** Average eye velocity traces of 14 participants between -100 and 100 ms from the pursuit latency. **c,d** Pattern dissimilarity of EEG activity between upper and lower tracking trials aligned by the visual motion onset. The color-shaded areas denote standard errors. The colored lines at the bottom of c and d show the time points where Mahalanobis distance was significantly different from zero (two-sided cluster-based permutation test, n = 14, cluster-defining threshold $p < 0.01$, corrected significance level $p < 0.01$, 50000 permutations).

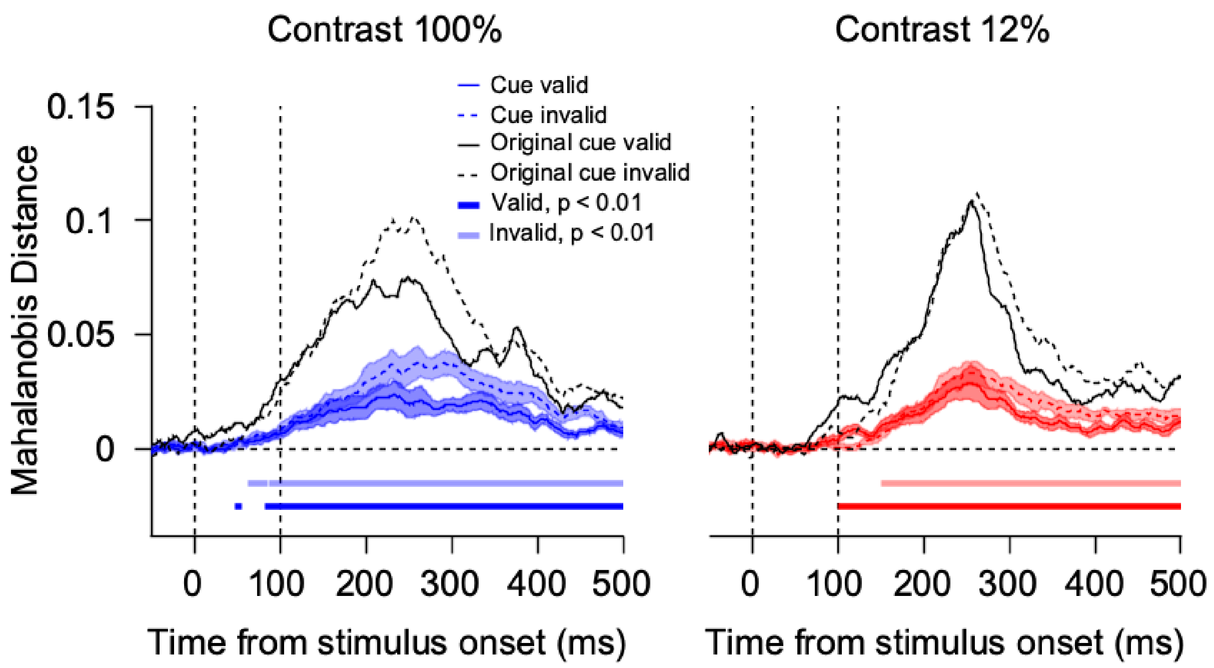

Supplementary Figure 3. Multivariate EEG pattern dissimilarity (Mahalanobis distance) between central and upper/lower tracking trials (blue and red lines) when the stimulus contrast was 100% (left) and 12% (right). The colored (blue and red) solid and dashed lines showed the EEG dissimilarity when the cue was valid and invalid. The color-shaded area denotes the standard error. The dark-colored line (valid) and light-colored line (invalid) at the bottom of each plot show the time points where Mahalanobis distance was significantly different from zero (two-sided cluster-based permutation test, n = 14, cluster-defining threshold $p < 0.01$, corrected significance level $p < 0.01$, 50000 permutations). The black solid and dashed lines show the EEG dissimilarity between the upper and lower trials from Figure 2a and b.

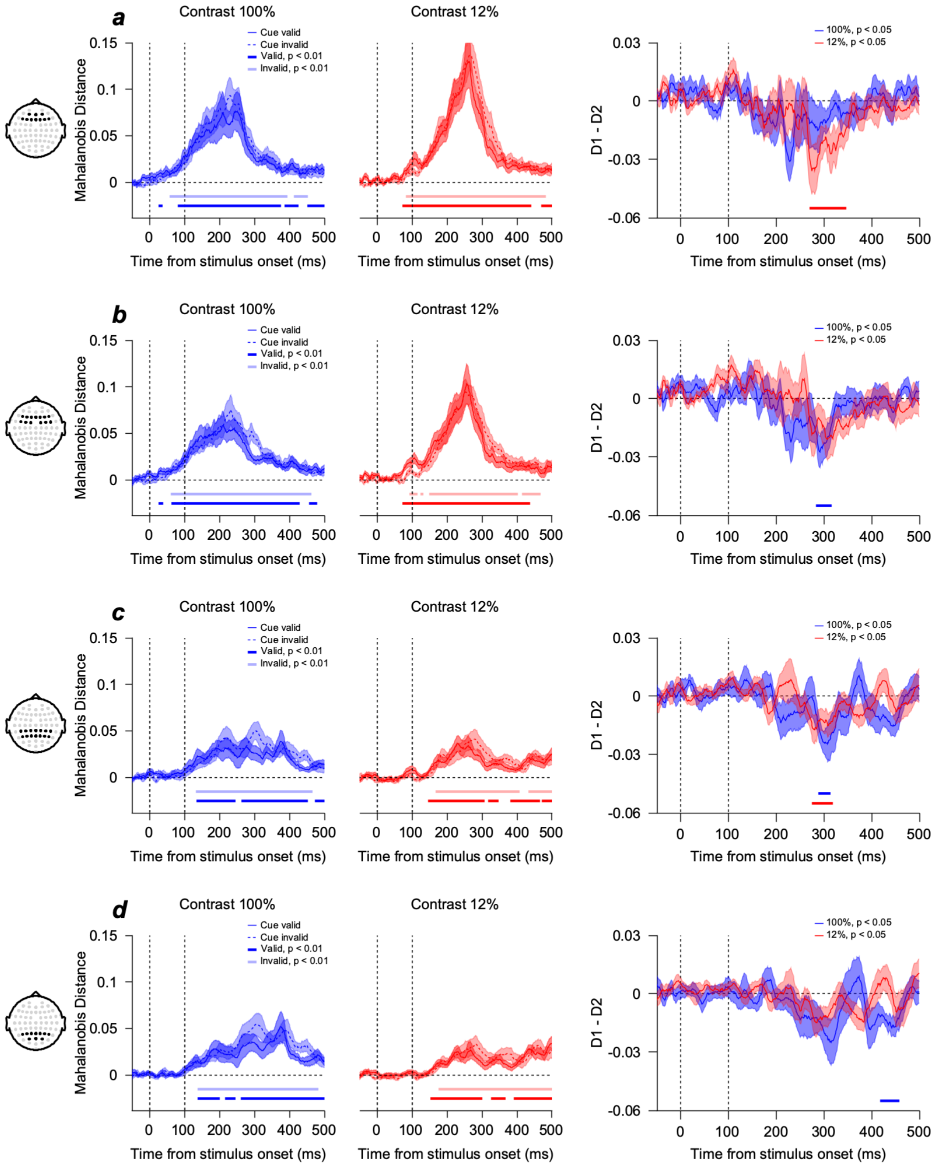

Supplementary Figure 4. a-d Multivariate EEG activity pattern dissimilarity (Mahalanobis distance) between upper and lower tracking trials from **a** Anterior-frontal channels (AFz, AF3, AF4, Fz, F1, F2, F3, F4, F5, and F6), **b** Frontal-central channels (Fz, F1, F2, F3, F4, F5, F6, FCz, FC1, FC2, FC3, FC4, FC5, and FC6), **c** Central-parietal channels (CPz, CP1, CP2, CP3, CP4, CP5, CP6, Pz, P1, P2, P3, P4, P5, and P6), **d** Parietal-occipital channels (Pz, P1, P2, P3, P4, P5, P6, POz, PO3, and PO4). The figure format is the same as Figure 4. The blue and red lines at the bottom of each plot (right column) show the significant time points (two-sided cluster-based permutation test, n = 14, cluster-defining threshold $p < 0.05$, corrected significance level $p < 0.05$, 50000 permutations). The dark-colored line (valid) and light-colored line (invalid) at the bottom of each plot (left and center column) show the significant time points (two-sided cluster-based permutation test, n = 14, cluster-defining threshold $p < 0.01$, corrected significance level $p < 0.01$, 50000 permutations). The color-shaded areas denote standard errors.

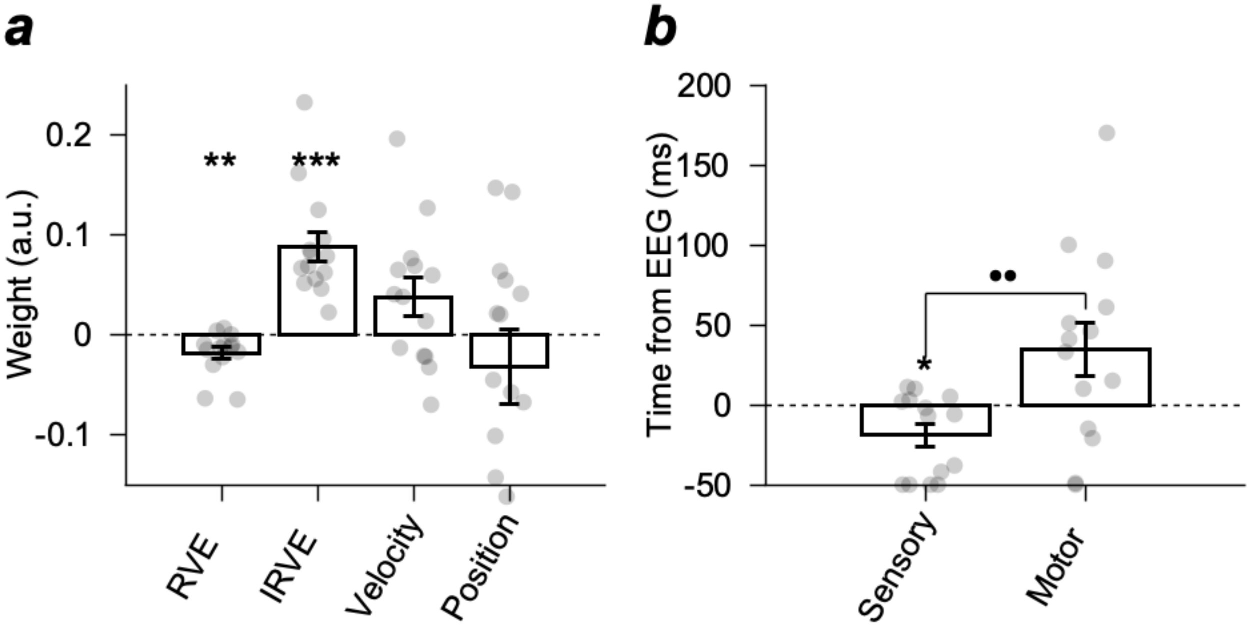

Supplementary Figure 5. Estimated parameters of the full model. a Averages of estimated weights for the dissimilarities in the retinal velocity error, integrated retinal velocity error, eye velocity, and eye position. **b** Averages of estimated times relative to EEG time for sensory inputs and motor outputs. The error bars denote the standard errors. $* p < 0.05$, $** p < 0.01$, $*** p < 0.001$, one-sample t-test; •• $p < 0.01$, two-sample t-test.

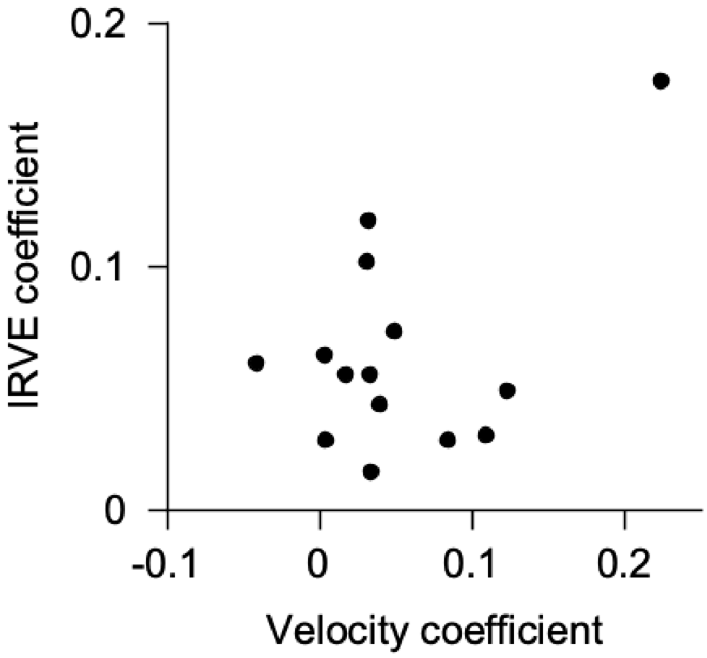

Supplementary Figure 6. A scatter plot of estimated parameters for the IRVE and the eye velocity of each participant (n = 14). There was no significant correlation between the weights of the IRVE and the eye velocity across participants (r = -0.1033, $p = 0.7270$).
