# Supplementary material for: Multivariate EEG activity reflects the Bayesian integration and the integrated Galilean relative velocity of sensory motion during sensorimotor behavior
Source: Commun Biol. 2023 Jan 28;6:113. doi: 10.1038/s42003-023-04481-2 (PMC9884247; doi:10.1038/s42003-023-04481-2)
Supplement: Supplementary file 3 — Description of Additional Supplementary Data [file 42003_2023_4481_MOESM3_ESM.docx]

**Description of Additional Supplementary Files**

**File name:** Supplementary Data 1

**Description:** The source data behind bar graphs (Fig. 1f inset, Fig. 3a inset, Fig. 6b-c, Supplementary Fig. 1a-b, Supplementary Fig. 5a-b) in the paper.
