# Supplementary material for: Multivariate EEG activity reflects the Bayesian integration and the integrated Galilean relative velocity of sensory motion during sensorimotor behavior
Source: Commun Biol. 2023 Jan 28;6:113. doi: 10.1038/s42003-023-04481-2 (PMC9884247; doi:10.1038/s42003-023-04481-2)
Supplement: Supplementary file 5 — Reporting Summary-New [file 42003_2023_4481_MOESM5_ESM.pdf]

nature research

Corresponding author(s): Joonyeol Lee

Last updated by author(s): Dec 27, 2022

Reporting Summary

Nature Research wishes to improve the reproducibility of the work that we publish. This form provides structure for consistency and transparency in reporting. For further information on Nature Research policies, see our Editorial Policies and the Editorial Policy Checklist.

Statistics

For all statistical analyses, confirm that the following items are present in the figure legend, table legend, main text, or Methods section.

n/a	Confirmed	
☐	☒	The exact sample size (*n*) for each experimental group/condition, given as a discrete number and unit of measurement
☐	☒	A statement on whether measurements were taken from distinct samples or whether the same sample was measured repeatedly
☐	☒	The statistical test(s) used AND whether they are one- or two-sided<br>*Only common tests should be described solely by name; describe more complex techniques in the Methods section.*
☐	☒	A description of all covariates tested
☐	☒	A description of any assumptions or corrections, such as tests of normality and adjustment for multiple comparisons
☐	☒	A full description of the statistical parameters including central tendency (e.g. means) or other basic estimates (e.g. regression coefficient) AND variation (e.g. standard deviation) or associated estimates of uncertainty (e.g. confidence intervals)
☐	☒	For null hypothesis testing, the test statistic (e.g. F, t, r) with confidence intervals, effect sizes, degrees of freedom and P value noted<br>*Give P values as exact values whenever suitable.*
☒	☐	For Bayesian analysis, information on the choice of priors and Markov chain Monte Carlo settings
☒	☐	For hierarchical and complex designs, identification of the appropriate level for tests and full reporting of outcomes
☒	☐	Estimates of effect sizes (e.g. Cohen's *d*, Pearson's *r*), indicating how they were calculated

Our web collection on statistics for biologists contains articles on many of the points above.

Software and code

Policy information about availability of computer code

Data collection	MAESTRO (https://sites.google.com/a/srscicomp.com/maestro/), BrainVision Recorder (https://brainvision.com/products/recorder/).
Data analysis	Matlab 2019a (Mathworks, Inc.), EEGLab (2019, 2021), FieldTrip toolbox (http://www.fieldtriptoolbox.org)

For manuscripts utilizing custom algorithms or software that are central to the research but not yet described in published literature, software must be made available to editors and reviewers. We strongly encourage code deposition in a community repository (e.g. GitHub). See the Nature Research guidelines for submitting code & software for further information.

Data

Policy information about availability of data

All manuscripts must include a data availability statement. This statement should provide the following information, where applicable:

- Accession codes, unique identifiers, or web links for publicly available datasets
- A list of figures that have associated raw data
- A description of any restrictions on data availability

The data and the code supporting this study's findings can be accessed from the link, https://semoconlab.com/codes/reliability-weighted-information-integration/, where preprocessed EEG and eye datasets were included.

Field-specific reporting

Please select the one below that is the best fit for your research. If you are not sure, read the appropriate sections before making your selection.

☐ Life sciences ☒ Behavioural & social sciences ☐ Ecological, evolutionary & environmental sciences

For a reference copy of the document with all sections, see nature.com/documents/nr-reporting-summary-flat.pdf

Behavioural & social sciences study design

All studies must disclose on these points even when the disclosure is negative.

Study description	quantitative experimental
Research sample	Healthy volunteers with normal or corrected to normal vision (mostly Sungkyunkwan University undergraduates and graduates), 16 male among 23 participants, age range between 20 and 31 yrs.
Sampling strategy	To obtain sufficient number of trials for multivariate EEG activity pattern, we asked for three visits per each participant, which allowed us to collect minimum of 2300 trials from each participant (Total number of conditions used in the experiment was 12(3 by 2 by 2)).
Data collection	Researcher was not blind to the purpose of the experiment. The horizontal and vertical eye positions and velocities of each participant were recorded using an infrared eye tracker (EyeLink 1000 Plus, SR Research). EEG data were recorded using 64-channel active electrodes (actiCAP, Brain Products, GmbH) and an EEG amplifier (BrainAmp, Brain Products, GmbH).
Timing	Data collection time range: March, 2017 ~ March, 2018.
Data exclusions	Among 17 human participants' EEG data, 1 participant's data were excluded because of poor EEG recording quality (more than 50% of ICs were detected as artifacts), and 2 were exluded because only two days' data were collected (insufficient trials).
Non-participation	Among 23 human participants, 6 were excluded after initial training session due to the poor eye movements (more than 30% of trials contained saccdic eye movements).
Randomization	Participants were not allocated into experimental groups.

Reporting for specific materials, systems and methods

We require information from authors about some types of materials, experimental systems and methods used in many studies. Here, indicate whether each material, system or method listed is relevant to your study. If you are not sure if a list item applies to your research, read the appropriate section before selecting a response.

Materials & experimental systems

n/a	Involved in the study	
☒	☐	Antibodies
☒	☐	Eukaryotic cell lines
☒	☐	Palaeontology and archaeology
☒	☐	Animals and other organisms
☐	☒	Human research participants
☒	☐	Clinical data
☒	☐	Dual use research of concern

Methods

n/a	Involved in the study	
☒	☐	ChIP-seq
☒	☐	Flow cytometry
☒	☐	MRI-based neuroimaging

Human research participants

Policy information about studies involving human research participants

Population characteristics	See above
Recruitment	Participants were recruited by the online advertisements
Ethics oversight	Sungkyunkwan University Institutional Review Board

Note that full information on the approval of the study protocol must also be provided in the manuscript.
